# Saving a Mandibular Molar With Distal Root Resorption: A Case Report

**DOI:** 10.7759/cureus.77775

**Published:** 2025-01-21

**Authors:** Abdulaziz A AlHelal, Faisal A AlHelal, Mohammed F Alem, Abdullah M Albaiz, Mohammed Y AlGammlas, Khalid W AlRashed, Mohammed Alselmi

**Affiliations:** 1 Prosthetic Dental Sciences, College of Dentistry, King Saud University, Riyadh, SAU; 2 College of Dentistry, King Saud University, Riyadh, SAU

**Keywords:** ceramic crown, endodontic treatment, external inflammatory root resorption, fiber post, indirect restoration, prosthodontics

## Abstract

In this case study, an all-ceramic (Emax) crown and a post and core restoration are used to successfully treat external inflammatory root resorption (EIRR) in a mandibular molar. A 21-year-old male patient was diagnosed with EIRR and hypercementosis after presenting with pain and significant damage to his lower right first molar. The patient chose a post and core restoration over extraction and implant placement due to budgetary constraints. Endodontic retreatment, fiber post cementation, core buildup, and Emax crown placement were all part of the treatment. At the one-year follow-up, the patient had a healed periapical region, healthy gingiva, and sound tooth structure. In managing EIRR cases, this case study emphasizes the importance of meticulous treatment planning and execution, focusing on selecting the best course of action for the long-term stability and health of the affected tooth. Both the patient's overall dental health and the expertly crafted restoration contributed to the favorable outcome.

## Introduction

It can be challenging to restore teeth that have had root canal therapy, especially if a sizable section of the tooth structure is gone. A core build-up is frequently required to assist with the rehabilitation that follows this significant loss. The amount of tooth structure that remains, the tooth's position in the mouth, and functional and aesthetic needs should all be taken into consideration when choosing the best course of action following root canal therapy [1]. Inflammations that develop in the tissues around a tooth's root are known as endodontic lesions or periapical lesions. These are usually brought on by bacteria getting inside the tooth, which frequently happens as a result of wear and tear, cavities, or trauma. Hypercementosis and external inflammatory root resorption (EIRR) are two of the problems that these lesions might cause [2].

EIRR is a condition in which odontoclasts, which are cells, destroy the surface of a tooth's root. This frequently occurs following dental trauma, which can weaken the tissues around the tooth and expose the root. Exposure of the root surface increases its susceptibility to bacterial infection and additional harm [3]. Endodontic retreatment could be required if the first endodontic treatment is unable to eradicate the infection or stop reinfection. The process entails removing the previous filling material, washing and sanitizing the tooth's interior, and then re-filling and sealing it [4]. To repair the tooth's structural integrity and offer retention for the final restoration, post and core restorations could be required in some situations [5]. Because it guarantees the restoration's long-term durability, properly cementing the post is essential [6].

Because of its superior aesthetics, great fracture resistance, and biocompatibility, Emax crowns, a type of all-ceramic restoration, have grown in popularity [7]. Because they can offer a robust and long-lasting restoration while maintaining the native tooth structure, they are frequently utilized on teeth that have undergone root canal therapy [7].

This case study outlines the sequential steps necessary for the successful management and follow-up of a young male patient with compromised endodontic treatment, hypercementosis in the middle third of the mesial root, and EIRR in the apical third of the distal root of a mandibular first molar.

## Case presentation

**Figure 1:**
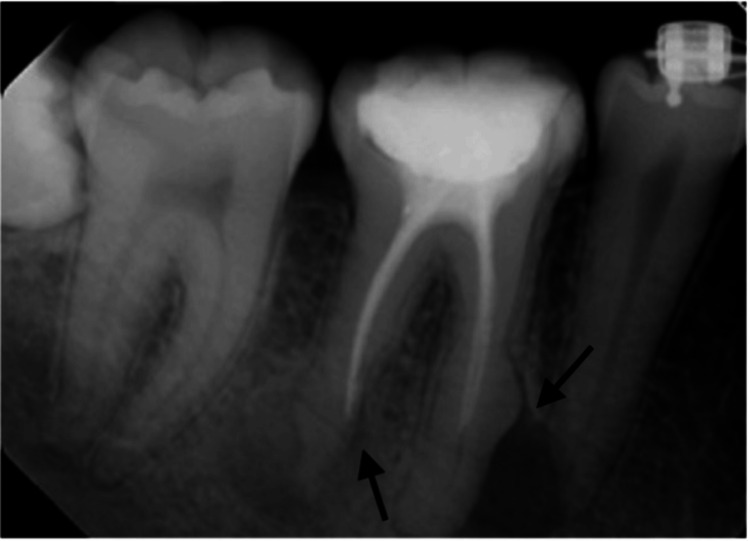
Preoperative periapical radiograph showing periapical lesions with mesial hypercementosis and external root resorption of the distal root.

A 21-year-old male patient arrived at the endodontic department of King Saud University Dental Hospital, complaining of pain around his lower right first molar. The patient did not report any medications or health issues. Clinical and radiographic evaluation revealed substantial damage to the lower right first molar (Figure 1).

**Figure 2:**
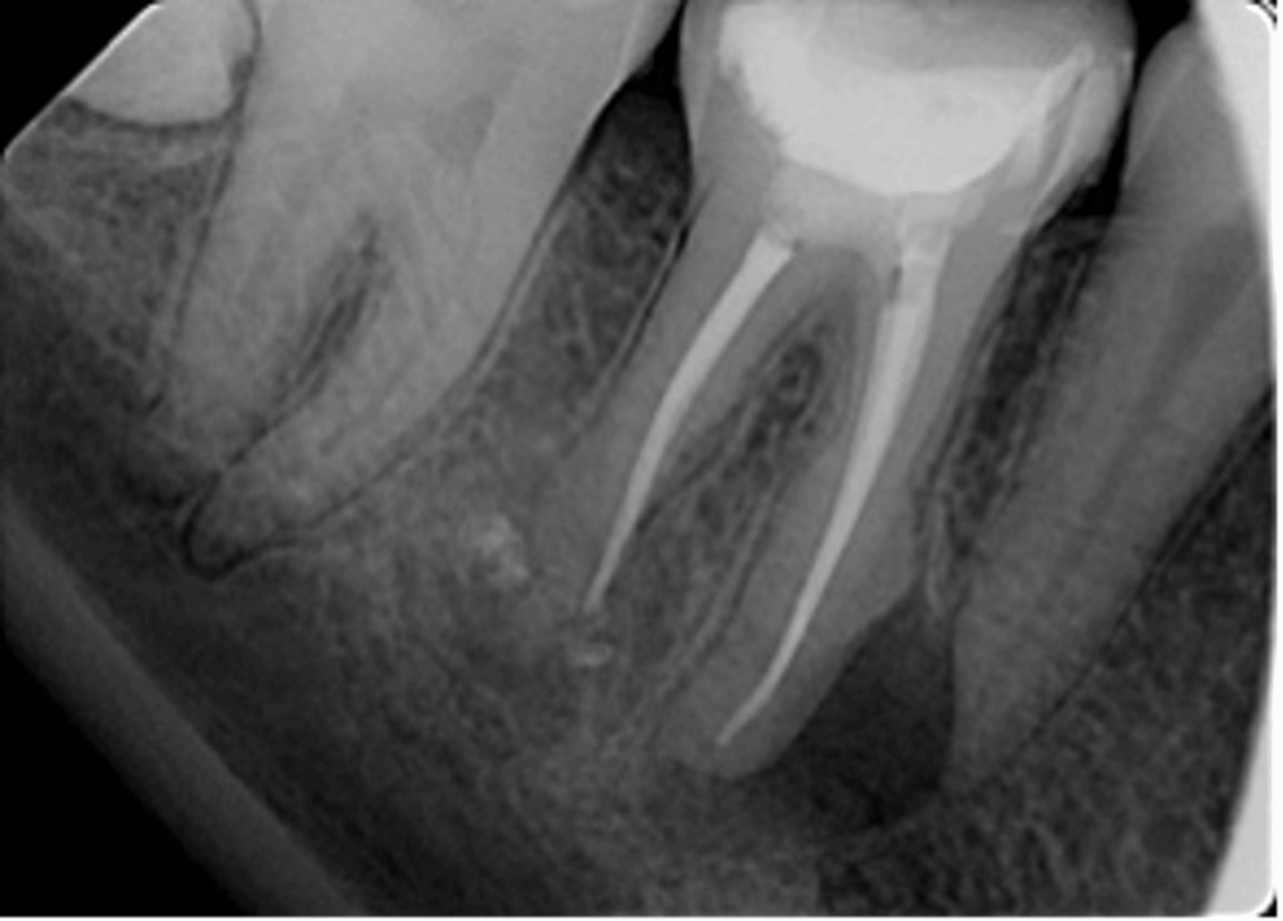
Post-endodontic retreatment periapical radiograph.

A periapical lesion was present, showing a combination of hypercementosis in the middle third of the mesial root and EIRR in the apical third of the distal root. An endodontic examination and diagnosis indicated that the patient had previously received treatment for apical periodontitis. The prognosis was uncertain due to the level of root resorption and tooth disintegration, although the tooth was restorable. A post and core procedure in the mesio-buccal canal, followed by a crown, was the first of several treatment options offered to the patient. The second, more promising alternative was to extract the tooth and replace it with an implant-retained crown. Due to financial constraints and a shorter treatment duration, the patient preferred the first option. The patient was referred to endodontics for root canal re-treatment and then returned for the final crown (Figure 2).

**Figure 3:**
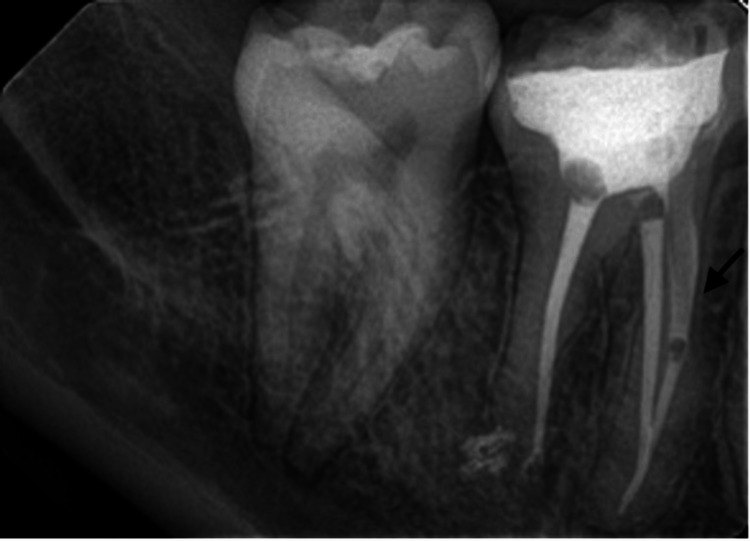
Periapical radiograph after placement of the fiber post and core build-up.

The endodontist recommended using the mesio-buccal canal rather than the distal canal for the post, prior to starting the post and core procedure, due to the root resorption on the mesial surface of the distal root. The clinical examination of the crown revealed a compromised distal wall and no facial wall. The mesial canal's working length was 24 mm, with 5 mm set aside as the apical seal. There was lingual curvature at a depth of 17 mm during post space preparation with Gates-Glidden and a universal post drill in the mesio-buccal canal, so the post's final depth was 17 mm, leaving a 7 mm apical seal. A glass fiber post (3M™ RelyX™ Fiber Posts Size 1 Yellow) was used, which matched the diameter of the post space preparation. The post was cemented using self-etch/self-adhesive dual-cure cement (3M™ RelyX™ Unicem Aplicap™ Self-Adhesive), and the tooth surface was acid-etched using acid etch (3M™ Scotchbond™ Universal Etching Gel 32% phosphoric acid; 3M Company, Saint Paul, Minnesota, United States) for 15 seconds, then rinsed and dried for an additional 15 seconds. Next, an adhesive (3M™ Scotchbond™ Universal Adhesive) was applied, air-blown for five seconds, and light-cured for 20 seconds, followed by core build-up (IVOCLAR VIVADENT MultiCore Flow Core Build-Up) (Figure 3).

The teeth were assessed for shade selection, and they were kept wet during the process using the traditional Vita shade guide (Vita Zahnfabrik, Bad Säckingen, Germany). Dental photographs were taken and sent to the laboratory, and A3.5 was the final shade chosen. The preparation was performed according to the guidelines published by the Canadian Dental Association. The crown preparation began with achieving 2 mm of occlusal clearance. Blue tapered chamfer diamond burs were used during the crown preparation. The margin for the crown preparation was a chamfer finish line with a 1 mm enamel margin all around. The axial walls were prepared using a tapered diamond bur.

After crown preparation, an impression was taken using polyvinyl siloxane (PVS) regular and light body (3M Express XT Regular Body and Light Body; 3M Company). A provisional restoration was placed using the direct provisional technique (3M ESPE Protemp 4 Dental Temporary Crown & Bridge), followed by cementation of the provisional crown using temporary cement (Temp-Bond Non-Eugenol (NE) by Kerr). The lithium disilicate crown was fabricated using a heat-press technique (IPS Emax Press; Ivoclar Vivadent, AG Schaan, Liechtenstein). A cast was made from the PVS impression, and a wax pattern was developed to create the mold. The mold was then used to fabricate the final all-ceramic restoration using a heat-pressed ceramic ingot.

**Figure 4:**
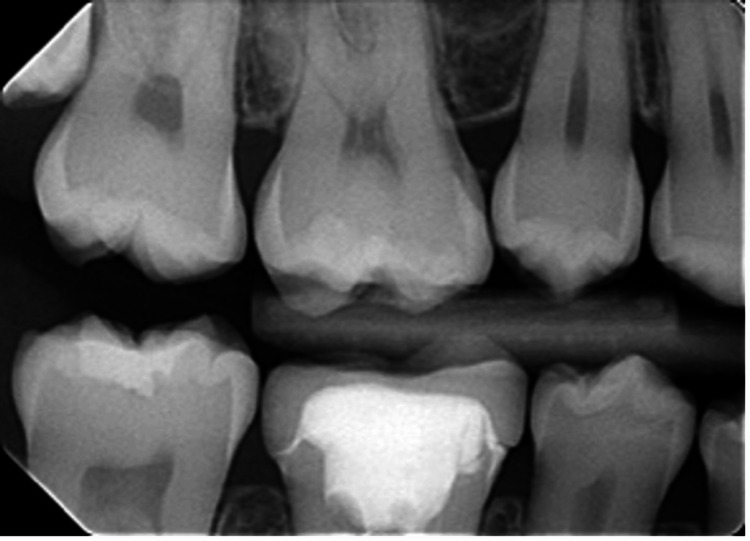
Bitewing radiograph after post crown cementation.

During the cementation visit, the temporary crown was carefully removed, and the prepared tooth was cleaned first with a sharp explorer, followed by cleansing with pumice and a brush. A final check for any leftover temporary cement was performed and removed if found. Buffered hydrofluoric acid gel (9.5%) (BISCO, Inc., Schaumburg, Illinois, United States) was applied to the crown's intaglio surface for 20 seconds, then rinsed and air-dried. A silane coupling agent (Bis-Silane Porcelain Primer; BISCO, Inc.) was applied to the crown's fitting surface, which had been acid-etched. The silane coupling agents were mixed in a 1:1 ratio. The first coat was placed on the etched internal surface of the lithium disilicate crown for 1 minute, followed by a second coat, which was applied for 30 seconds and then air-dried with an air syringe. A self-etch/self-adhesive dual-cure cement (Maxcem Elite™ Universal Resin Cement; KaVo Kerr, Brea, California, United States) was applied to the internal surface of the crown, and the crown was placed on the prepared tooth. No excess cement was left; an explorer was used on the buccal and lingual surfaces, and dental floss was used on the interproximal surfaces. The cement was then cured for 20 seconds on all surfaces of the tooth. A bite-wing radiograph was taken to ensure the complete removal of any cement remnants (Figure 4).

**Figure 5:**
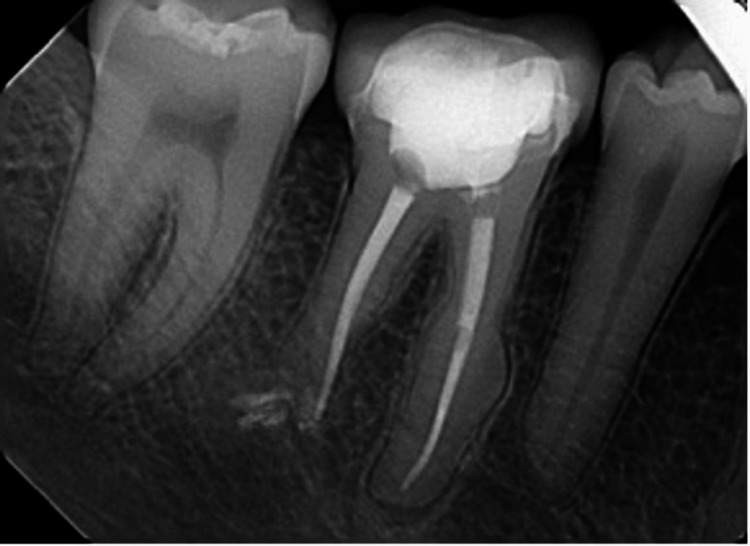
Periapical radiograph after one-year follow-up.

Occlusion was checked with articulating paper before and after cementation to identify any unwanted interference. Shimstock (Bausch Arti Fol Metallic Shimstock Film 12µ; Dr. Jean Bausch GmbH & Co. KG) was used to assess occlusal contacts on all teeth and confirm the centric occlusion of the crown. One week after crown cementation, the patient was re-evaluated, and the integrity of the crown was examined; no issues were observed. About a year later, the patient was referred for a follow-up visit (Figure 5).

The patient then presented with a healed periapical region and a healthy periodontium. Appointments for additional follow-up were scheduled.

## Discussion

Dentists must carefully plan and execute their restorations in order to successfully restore molars impacted by EIRR. To guarantee the long-term stability and health of the impacted teeth, the best course of action must be chosen. Because they alter the structure and contour of the tooth, endodontic lesions, particularly those involving EIRR and hypercementosis, present specific challenges in restorative dentistry [8-11]. EIRR is a pathologic condition in which tooth-resorbing cells known as dentoclasts or odontoclasts cause the loss of cementum and/or dentin [10-12]. Periapical lesions, which are inflammatory reactions in the periradicular tissues brought on by bacterial invasion from the root canal system, are frequently linked to this process [10]. The presence of EIRR can influence post and core procedures, as the weakened tooth structure may necessitate careful consideration for post placement and cementation [13].

There are two primary phases in the formation of EIRR: stimulation and damage. The precementum and predentin, two non-mineralized tissues that protect the root, are harmed during the damage stage [5]. Numerous things, like as trauma, illness, and orthodontic treatment, can cause this damage. Multinucleated cells, like odontoclasts, can then colonize the exposed mineralized tissue, initiating the resorption process. These multinucleated cells are stimulated during the activation stage, which causes the tooth's hard structures to break down. Microorganisms in the root canal and dentinal tubules frequently cause this stimulation by releasing chemicals into the surrounding tissues. These chemicals trigger an immunological reaction, which results in the production of signaling molecules that encourage some immune cells to develop into odontoclasts [10].

Depending on the severity of EIRR, its clinical appearance can vary. It is only visible on radiographs in the early stages and may not cause any symptoms [10]. As it worsens, the tooth may become loose, bulge, and hurt [7,10]. EIRR can seriously harm the root in extreme situations, which may result in the tooth breaking or requiring extraction. In order to manage EIRR, a root canal is usually performed in order to eradicate the infection. Surgery may be required to remove or repair the injured section of the root in more severe cases if the damage is considerable. For teeth with EIRR, the long-term result is contingent upon a number of variables, such as the degree of resorption, any comorbidities, and the patient's overall oral health [10].

Before the tooth is utilized to support a dental bridge or other repair, these instances should ideally be observed for a minimum of 12 months. The risk of fracture, however, increases if a crown or other treatment is placed on the tooth later than necessary. Important things to think about are the bite forces on the tooth and the amount of healthy dental structure that is still there. Delaying the placement of a well-made restoration can result in treatment failure and have an impact on the tooth's long-term health [14]. Fiber posts are frequently used in restorative dentistry to help hold the final restoration in place and strengthen teeth that have undergone root canal therapy [6,15]. Fibers, such as carbon, glass, or quartz, are embedded in these posts [16].

The use of fiber posts in dentistry began in 1989 with the introduction of the composite post in France. These posts, initially made of carbon or graphite, revolutionized dentistry by offering a reliable alternative to metal posts [14]. Fiber posts offer aesthetic benefits, similar flexibility to natural dentin, ease of handling for dentists, and easy removal for root canal treatment, making them a more suitable alternative to traditional metal posts [15]. Fiber posts are effective in restoring teeth after root canal treatment, but potential issues include looseness, tooth fracture, or root damage. Cusp coverage is recommended for tooth protection. Intra-radicular posts in pulpless teeth provide support, but excessively long or short posts can weaken the root [1,15].

The strength and hardness of three distinct composite materials (FiltekTM Z350 XT, FiltekTM bulk fill, MultiCore Flow) used for tooth restoration were examined in a study. According to the investigation, each material has distinct qualities. Filtek™ bulk fill was the most flexible, and Filtek™ Z350 XT was the strongest and hardest. Despite being the least reliable of the three, MultiCore Flow had the greatest Weibull modulus, a gauge of dependability [17]. Because of these advantages, multicore was used as it was pertinent to the current scenario of our case report. A study by Mounajjed et al. compared two methods of Emax lithium disilicate restorations: pressing and CAD/CAM [18,19]. The pressing technique resulted in a more accurate fit with smaller gaps. A study on Ipsen's Pressable System e.max all-ceramic restorations showed that 94% of restorations were successful after five years, with glass-ceramic crowns and bridges having a 90-95% success rate [20].

The etiology of EIRR determines how it is treated. Endodontic therapy, which includes the use of intra-canal medications, is necessary to eliminate the microorganisms causing infection-induced or infection-sustained tooth resorption. This is followed by a permanent restoration that is in harmony with the surrounding dentition, preventing any microorganisms from causing infection through the coronal part of the tooth. This approach helps rebuild the resorbed tooth structure and ensures the long-term success of the restored tooth.

## Conclusions

The case report discusses the successful management of a mandibular molar affected by EIRR using a post-and-core restoration followed by an all-ceramic crown. The patient chose this treatment due to financial constraints and a shorter treatment time, despite the better prognosis offered by extraction and implant placement. The report emphasizes the importance of careful treatment planning, considering tooth structure, bite forces, and fracture risk when determining the timing of crown placement. The success of the case at the one-year follow-up was attributed to optimal endodontic treatment, a well-made restoration, and the patient's overall oral health.
